# Discrimination between healthy and patients with Parkinson’s disease from hand resting activity using inertial measurement unit

**DOI:** 10.1186/s12938-021-00888-2

**Published:** 2021-05-22

**Authors:** Luciano Brinck Peres, Bruno Coelho Calil, Ana Paula Sousa Paixão Barroso da Silva, Valdeci Carlos Dionísio, Marcus Fraga Vieira, Adriano de Oliveira Andrade, Adriano Alves Pereira

**Affiliations:** 1 Postgraduate Program in Electrical and Biomedical Engineering, Faculty of Electrical Engineering, Centre for Innovation and Technology Assessment in Health, Federal University of Uberlândia, Uberlândia, Brazil; 2 Department of Information Technology, UNA Uberlândia University Center, Uberlândia, Brazil; 3 Department of Morphology, Mineiros University Center, Mineiros, Brazil; 4 Faculty of Physical Education and Physiotherapy, Federal University of Uberlândia, Uberlândia, Brazil; 5 Bioengineering and Biomechanics Laboratory, Federal University of Goiás, Goiânia, Brazil

**Keywords:** Parkinson disease, Inertial sensors, Classifiers, Rest tremor

## Abstract

**Background:**

Parkinson’s disease (PD) is a neurological disease that affects the motor system. The associated motor symptoms are muscle rigidity or stiffness, bradykinesia, tremors, and gait disturbances. The correct diagnosis, especially in the initial stages, is fundamental to the life quality of the individual with PD. However, the methods used for diagnosis of PD are still based on subjective criteria. As a result, the objective of this study is the proposal of a method for the discrimination of individuals with PD (in the initial stages of the disease) from healthy groups, based on the inertial sensor recordings.

**Methods:**

A total of 27 participants were selected, 15 individuals previously diagnosed with PD and 12 healthy individuals. The data collection was performed using inertial sensors (positioned on the back of the hand and on the back of the forearm). Different numbers of features were used to compare the values of sensitivity, specificity, precision, and accuracy of the classifiers. For group classification, 4 classifiers were used and compared, those being [Random Forest (RF), Support Vector Machine (SVM), K-Nearest Neighbor (KNN), and Naive Bayes (NB)].

**Results:**

When all individuals with PD were analyzed, the best performance for sensitivity and accuracy (0.875 and 0.800, respectively) was found in the SVM classifier, fed with 20% and 10% of the features, respectively, while the best performance for specificity and precision (0.933 and 0.917, respectively) was associated with the RF classifier fed with 20% of all the features. When only individuals with PD and score 1 on the Hoehn and Yahr scale (HY) were analyzed, the best performances for sensitivity, precision and accuracy (0.933, 0.778 and 0.848, respectively) were from the SVM classifier, fed with 40% of all features, and the best result for precision (0.800) was connected to the NB classifier, fed with 20% of all features.

**Conclusion:**

Through an analysis of all individuals in this study with PD, the best classifier for the detection of PD (sensitivity) was the SVM fed with 20% of the features and the best classifier for ruling out PD (specificity) was the RF classifier fed with 20% of the features. When analyzing individuals with PD and score HY = 1, the SVM classifier was superior across the sensitivity, precision, and accuracy, and the NB classifier was superior in the specificity. The obtained result indicates that objective methods can be applied to help in the evaluation of PD.

**Supplementary Information:**

The online version contains supplementary material available at 10.1186/s12938-021-00888-2.

## Background

Parkinson’s disease is the second most common neurodegenerative disease; however, the epidemiological data are not determined precisely [1]. This difficulty can be explained by the different criteria used across the different studies and the precision of the PD diagnosis [1, 2]. There is a common acceptance that PD possesses a prevalence of approximately 1–2% of the population over 65 years of age and 0.3% of the general population [1]. It was estimated that in 2020, PD affected more than 9 million individuals worldwide [3].

The cause of PD is unknown. However, various factors can be considered as being of risk for PD, which include gender (women are marginally more likely to be affected due to longevity), ethnicity (PD possesses greater prevalence in Europe and in North America), genetics, exposure to toxic substances, lethargic encephalitis sequelae, head trauma, and emotional stress [2]. Nevertheless, the main factor of risk is age [4], and as such, it is expected that there will be a drastic increase over the coming years due to the aging of the population [1]. The symptoms of PD can be non-motor or motor. The non-motor symptoms include neuropsychiatric characteristics, dysautonomia, sleep disorder, sensory dysfunction, pain, and fatigue [1]. The motor symptoms that may appear are muscle stiffness, bradykinesia, tremors, and postural imbalance [5], where resting tremor is presented as the main motor symptom (in around 70% of individuals) [6]. Therefore, PD can have serious impacts on the social and personal life of the patient, which can include incapacity of feeding oneself, drinking water, writing, walking, and even speaking [7].

The impact of PD on life quality, in the decrease in capacity when performing daily routine activities added to prevalence, makes the correct diagnosis of PD essential to outline treatment and measures that can alleviate symptoms, thus improving the ability of individuals to resume their normal daily activities [8]. The correct evaluation of PD can assist clinicians in corrective interventions and improve the life quality of individuals with PD [9], and thus, there are tools for assessing the current stage of PD through use of the Hoehn and Yahr scores [10]. The diagnosing of PD is not a simple task, as the diagnosis can suffer alterations due to the age of the individual and the evolution of symptoms [11]. The most commonly used tool for diagnosing PD is the *Unified Parkinson’s Disease Rating Scale* (UPDRS) [12]. The UPDRS combines a series of clinical scales and questionnaires that have the objective of evaluating the presence and progress of PD motor symptoms [13]. However, the use of UPDRS is more dependent on the professional and, as such, is performed at a certain expense of the patient, as the experience of the professional ends up being a decisive factor when presenting the symptoms at the moment of the diagnosis. In addition, the symptoms of PD can vary due to a number of factors, such as mood, diet, daily habits of patient, absence or not of medication for treating symptoms, and age [14]. One solution to these limitation found in UPDRS could be the use of objective methods [15].

The correct diagnosis of PD is vital for controlling the symptoms and improving the life quality of patients. Although being the most widely used, the UPDRS does have its limitations, due to the time consumed in its application and its high degree of subjectivity [12]. An example of the said subjectivity is presented in studies that show a tendency of evaluators to underestimate the severity of the tremor on the least affected side, when the other side presents a severe tremor; this tendency is attributed to less attention being given to the least affected side [16]. The use of an objective method minimizes the need for an experienced professional being involved in the diagnosis process, as it is not always that the patients have such a professional at hand. The challenge takes on greater proportions when assessing individuals with PD in the initial stages of the disease, as is the case of the group of individuals with PD in this study. According to [17], UPDRS is not suitable for assessing PD in its early stages, as most items are related to the more advanced stages of PD.

Due to the aforementioned, there arises the need to discover methods capable of diagnosing PD in a more objective manner. In this sense, the literature presents some proposals using objective methods for the diagnosis of PD [18]. Among these methods, emphasis is placed on those related to the evaluation of movement. This preference is justified by the fact that the symptoms and results of the treatment are manifested through movement in the form of the tremor, bradykinesia, and dyskinesia [7]. The analysis of movement by means of inertial sensors (accelerometers, gyroscopes, and magnetometers) is quite widespread, due to their size and low cost, this allows such instruments to be easily assembled and positioned on different parts of the human body [7].

From the inertial data, one can extract features that will be used for the diagnosis of PD. Currently, there exists a number of studies that propose methods for a more objective diagnosis of PD, using inertial sensors and classifiers. The use of predictors, such as random forest (RF), support vector machine (SVM), K-nearest neighbor (KNN), and Naive Bayes (NB), have helped to differentiate the PD tremor from the essential tremor.

These predictors have been applied to individuals with PD. Research conducted by Kuhner [12] proposes a method to objectively quantify the tremor in individuals with PD, using an RF classifier. The SVM classifier has been used in research to differentiate tremor at rest and postural tremor in individuals with PD from essential tremor in healthy individuals, in addition to being used in research to differentiate the various types of tremor present in individuals with PD [19–21]. The authors in [22] used the KNN for the recognition of daily activities of individuals with PD and healthy individuals, by means of features extracted from inertial sensors. The study conducted in [23] used the NB classifier to diagnose PD, using a PD databank from the UCI machine learning repository.

The above-mentioned classifiers, used in the examples of application concerning individuals with PD, are fed with data that are collected from sensors positioned at strategic points on the individual, through their performing of allocated tasks, but these can sometimes go on to be tiring and as such influence the classification. In this scenario, due to the challenges in diagnosing PD, mainly in the early stages, along with the subjectivity of the main diagnosis method, the introduction of an objective method using inertial sensors for the diagnosis of PD is an area worthy of investigation. Therefore, this study proposes the analysis of feature movements, extracted from inertial sensors, as a tool for diagnosing PD. Our proposal simply uses the hand at rest as a task. In addition, we aim to test the performance of different classifiers (RF, SVM, KNN, and NB) to find suitable methods to differentiate the PD group, in the early stages, from the healthy group.

## Results

Additional file 1, Figures S1–S3, illustrate the signals collected from the accelerometer, gyroscope, and magnetometer on the three axes (x, y and z) and the corresponding resultant signal.

The 18 features presented in Table 6 (see section “Methods”) were calculated from the signals originating from the sensors, in the same sequence that these are presented in Table 6. As such, a total of 108 features were calculated for the 6 sensors, distributed in the following way: 36 features for the accelerometers, 36 features for the gyroscopes, and 36 features for the magnetometers [the system is composed of two inertial measurement units (IMUs), each IMU possesses an accelerometer, a gyroscope, and a magnetometer].

For all individuals with PD, the highest values for the metric of accuracy were achieved with 10% of the set of features, and the highest values of the metrics sensitivity, specificity, and precision were achieved with 20% of the feature set. For individuals with PD and HY = 1, the highest values for the metric of specificity was achieved with 20% of the feature set, and the highest values of the metrics sensitivity, precision, and accuracy were achieved with 40% of the feature set. As such, Table 1 presents the features for 20% of the feature set used to feed the classifiers in the analysis of all individuals with PD (the highlight in bold represents 10% of the features used to feed the classifiers). In addition, Table 1 also presents the features for 40% of the set of features used to feed the classifiers in the analysis of individuals with PD and HY = 1 (the highlight in bold represents 20% of the characteristics used to feed the classifiers).

**Table 1:** Features for 20% and 40% of the feature set used to feed the classifiers in the analysis of all individuals with PD and individuals with PD and HY = 1, respectively

Sensors	Features	
	All individuals with PD (20% of the feature set)	Individuals with PD and HY = 1 (40% of the feature set)
Gyroscope 1		MAV
	Zero crossing	MAVFD
	F80^a^	MAVSD^b^
	EnAp	RMS^b^
	EnFuzzy	PICO^b^
	SKEW^a^	Zero crossing
	KURTOSIS^a^	VAR^b^
		RANGE^b^
		KURTOSIS
Gyroscope 2	Zero crossing^a^	Zero crossing^b^
	Fpico^a^	Fmedia^b^
	F80^a^	F50
	EnAp	F80^b^
	EnFuzzy	EnAp^b^
	SKEW	EnFuzzy^b^
	KURTOSIS	INTQ
Accelerometer 1		MAV
		MAVFD
		MAVSD
	F80	PICO^b^
	EnAp	Fpico^b^
	SKEW^a^	RANGE^b^
	**KURTOSIS**^a^	INTQ^b^
		SKEW^b^
		KURTOSIS^b^
		Power3.5–7.5^b^
Accelerometer 2		MAV
		MAVFD
		MAVSD^b^
	Zero crossing	RMS
	F80	Zero crossing^b^
	EnAp^a^	F80
	EnFuzzy^a^	EnAp
	KURTOSIS^a^	EnFuzzy
		INTQ
		Power3.5–7.5^b^
Magnetometer 1	No feature	F50^b^
		F80
		EnAp
		EnFuzzy
		VAR
		INTQ
Magnetometer 2	No feature	MAVFD
		INTQ^b^

^a^10% of the features in the analysis of all individuals with PD

^b^20% of the features in the analysis of individuals with PD and HY = 1

Noted on Table 1 is that according to the classification of the most important 20% of features, only two sensors (accelerometer and gyroscope) are sufficient to feed the classifiers in the analysis of all individuals with PD.

Table 2 shows the results obtained by the classifiers KNN, SVM, RF, and NB in terms of sensitivity, specificity, precision, and accuracy for 10% of the feature set and 20% of features for all individuals with PD. The parameters of the classifiers were defined experimentally and those that produced the best response in terms of sensitivity, specificity, precision, and accuracy were chosen. Hence, the classifiers were configured in the following way:

- KNN: K = 3;
- Random Forest: 120 trees;
- Naïve Bayes: kernel (normal);
- Support Vector Machine: polynomial kernel.

**Table 2:** Evaluation of the classifiers KNN, SVM, RF, and NB of all individuals with PD

Classifiers	Metrics				Training accuracy
	Sensitivity	Specificity	Precision	Accuracy	
10% of features					
KNN	0.833	0.667	0.800	0.769	0.810
SVM	0.833	0.733	0.833	**0.800**	0.810
RF	0.667	0.800	0.842	0.718	0.690
NB	0.542	0.800	0.813	0.641	0.667
20% of features					
KNN	0.750	0.667	0.783	0.718	0.738
SVM	**0.875**	0.600	0.778	0.769	0.738
RF	0.458	**0.933**	**0.917**	0.641	0.690
NB	0.500	0.867	0.857	0.641	0.667

The highest values of the metrics are highlighted in bold

From Table 2, one notes that the highest accuracy and sensitivity were achieved with the classifier SVM, fed with 10% and 20% of all features, respectively. The highest specificity and precision were achieved with the classifier RF, fed with 20% of all features.

For individuals with PD and HY = 1, the highest values for the metrics sensitivity, precision, and accuracy were achieved with the classifiers fed with 40% of the feature set and the highest values for the metric of specificity was achieved with the classifiers fed with 20% of the feature set. Table 3 shows the results obtained by the classifiers KNN, SVM, RF, and NB in terms of sensitivity, specificity, precision, and accuracy for 40% and 20% of the feature set.

**Table 3:** Evaluation of the classifiers KNN, SVM, RF, and NB of individuals with PD and with HY = 1

Classifiers	Metrics				Training accuracy
	sensitivity	Specificity	Precision	Accuracy	
20% of features					
KNN	0.667	0.556	0.556	0.606	0.722
SVM	0.733	0.611	0.611	0.667	0.639
RF	0.600	0.444	0.474	0.515	0.722
NB	0.533	**0.833**	0.727	0.697	0.583
40% of features					
KNN	0.733	0.556	0.579	0.636	0.778
SVM	**0.933**	0.778	**0.778**	**0.848**	0.750
RF	0.533	0.778	0.667	0.667	0.611
NB	0.667	0.722	0.667	0.697	0.583

The highest values of the metrics are highlighted in bold

The comparison of the values found on Tables 2 and 3 shows that when only individuals with PD with HY = 1 are considered, the performance of the classifiers is better in relation to the metrics sensitivity and accuracy, but the metrics specificity and precision are lower in relation to the group that uses all individuals with PD.

## Discussion

### Evaluation of extracted features

The signal that produces the inertial sensor recordings, arising from Parkinson’s disease, possesses various proposals for its evaluation. The signal from inertial sensor recordings in PD can be evaluated through features related to frequency [24, 25], and other features related to amplitude, signal entropy, form of data distribution, and variability can also provide important features when analyzing the signal from inertial sensor recordings in individuals with PD [24]. Amplitude is linked to a scalar measurement (negative or positive) in the oscillation of a movement [26]. Additionally, some studies show the use of entropy can aid in the classification process of other diseases [27].

There were 18 features chosen that allow for the classification of individuals as healthy or with PD. We used 5 features related to amplitude (RMS, PEAK, MAV, MAVFD, and MAVSD), 6 feature related to frequency (Zero Crossing, Fmedia, Fpico, F50, F80, and Power3.5_7.5), 2 features related to signal entropy (ApEn and FuzzyEn), 3 features related to signal variability (VAR, Range, and IntlA), and 2 features related to data distribution (Skewness and Kurtosis).

By analyzing all individuals with PD, the groups of features Amplitude and Variability were not considered important for the calculation of the metrics sensitivity, specificity, precision, and accuracy. The features of frequency, form of data distribution and entropy stood out in this differentiation in relation to sensitivity and accuracy. This observation was already expected to some extent, as participants with PD were in early stages and without any clear signs of tremor.

### Evaluation of classifiers

This study compared the KNN, SVM, RF, and NB classifiers, KNN is currently used as a benchmark for this type of application, and the SVM, RF and NB classifiers are widely used today, which means that this comparison can aid the PD diagnosis in becoming more effective [28–30].

The study performed in [31] also used the inertial sensors of accelerometer and gyroscope, but applied to gait of those with PD alongside healthy individuals, to differentiate the relative parameters for gait between the two groups. The classifiers used were also, among others, the KNN, SVM, RF, and NB, and the parameter used for validating the result was accuracy, the classifier that presented the best general result was the KNN with an accuracy of 84.5%. The collaborators in [32] analyzed the gait of individuals with PD alongside healthy individuals, by means of a force platform. The classifiers used in the present study were also used, among others, in the study by [32]. The best accuracy obtained by the authors was with the SVM classifier of around 90%, followed by the methods RF and KNN with an accuracy around 87%.

Our study agrees with that in [32], as in our study, the best performance in relation to accuracy, in terms of the comparison between classifiers was presented by SVM in both analysis, when analyzing all individuals with PD, along with the analysis of individuals with PD and HY = 1 (80% and 85% respectively).

The four statistical metrics used (sensitivity, specificity, precision, and accuracy) possess the capacity of demonstrating the performance of the classifiers. Our study showed a better performance for SVM classifier in the sensitivity and accuracy metrics. When analyzing all individuals with PD, the best performance was found in the RF classifier in relation to the specificity and precision metrics. When analyzing individuals with PD and HY = 1, the best performance was found in the NB classifier in relation to specificity and the best performance in relation to the precision metric was found in the SVM classifier.

The accuracy found in our study was similar that presented in the results of [31, 32]. Despite the metrics calculated in our work being close to the values found in [31, 32], it is important to highlight the statement of [33], where emphasis is placed in their work on the performance comparison of the classifiers among several studies, which must be carried out carefully, due to the differences involved in the calculation of the metrics, such as the parameters of the algorithms and the features used.

### Percentage of features

**Fig. 1:**
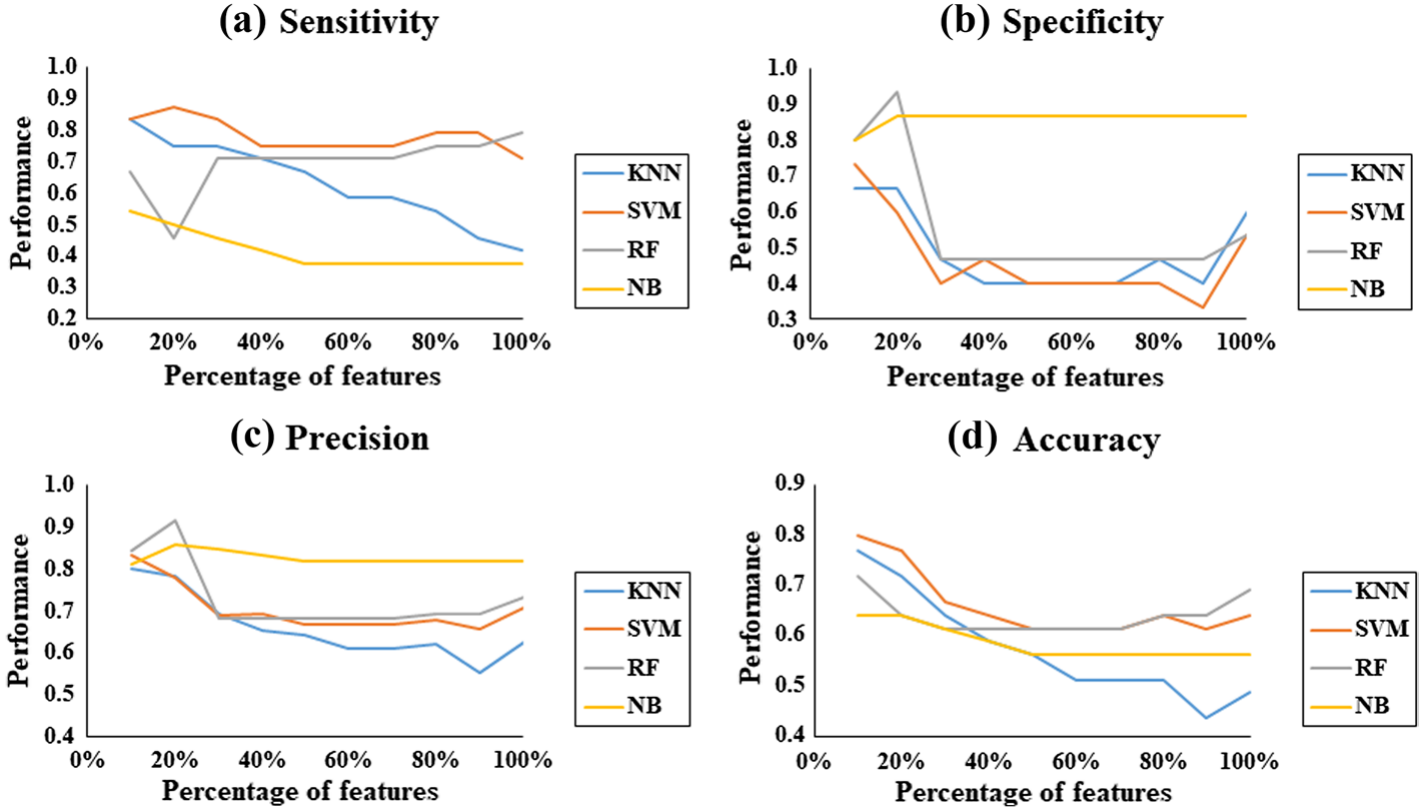
Classifier performance in relation to the number of features of individuals with PD with score HY = 1 and 2. Metrics: **a** sensitivity, **b** specificity, **c** precision, and **d** accuracy

Despite not being the objective of the article, the behavior of the classifiers in relation to the percentage of the features used was analyzed. Figure 1 shows the performance of the classifiers when all individuals with PD are analyzed. Figure 1 shows that when all individuals with PD were analyzed together, the best performance in relation to the metrics sensitivity and accuracy is presented by the SVM classifier, when using 20% and 10% of the feature set, respectively. Regarding the metrics specificity and precision, the best performance was presented by the RF classifier using 20% of the feature set, only features derived from the accelerometer and gyroscope sensors were needed for classification.

**Fig. 2:**
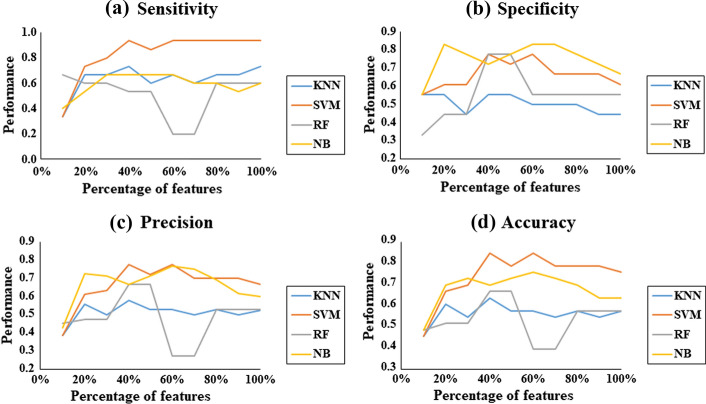
Classifier performance in relation to the number of features of individuals with PD and HY = 1. Metrics: **a** sensitivity, **b** specificity, **c** precision, and **d** accuracy

Figure 2 shows the performance of the classifiers with individuals suffering from PD with the score HY = 1 being analyzed. In this analysis, the best performance is presented by the SVM classifier in regards to the metrics sensitivity, precision, and accuracy, using 40% of the feature set. The best performance in relation to specificity was the NB classifier using 20% of the feature set, and all sensors were needed for classification (accelerometers, gyroscopes, and magnetometers).

### Evaluation of the results for the PD groups

Two groups of individuals with PD were evaluated in comparison to the healthy group, and the first group evaluated was composed of all individuals with PD (HY scores 1 and 2). The other group analyzed were individuals with PD with HY = 1. The results for the metrics sensitivity and accuracy were superior in the analysis of the group of individuals with HY = 1. This result can be explained by the presence of individuals with more evident tremor in the first analysis (HY = 2), these individuals possess features that are very much separate from the other participants in the PD group, leading to an approximation of the features between the healthy group and the individuals with PD that present lesser oscillations measured from the inertial sensors.

Noteworthy here is that the individuals in our sample showed signs of Parkinson`s in the early stages, usually stages that are difficult to diagnose. Success in differentiation suggests that these classifiers are promising for differentiating between individuals with PD from healthy individuals. Therefore, we encourage future studies that investigate the success rate of classifiers to differentiate the groups (PD and healthy) with large samples, as well as their ability to classify bradykinesia.

### Limitations

The main limitations of this study are the number of participants and the absence of a guideline to define the features used and the positioning of the IMUs. Furthermore, our work classified the groups of subjects with Parkinson's disease and the group of healthy subjects based on inertial sensors. The group of subjects with Parkinson's disease was in the early stages of the disease, so that 11 of the 15 subjects with Parkinson's disease did not show visible signs of tremor. However, the signals collected by the inertial sensors had oscillations that allowed for the classification of the analyzed groups. Thus, although 70% of the subjects with PD experience tremors [6], there are a number of people with PD who do not experience tremor or it is not the first symptom presented, in these cases, the technique presented is not able to classify the groups. In addition, the only symptom analyzed was the oscillations present in the signal, and other symptoms should be considered to improve the accuracy of the method for the proper classification of the groups studied.

## Conclusion

When we analyzed all patients with PD, our study suggests that for the calculation of accuracy in the evaluation of PD, only 11 features, comprising of accelerometers and gyroscopes, possess the best performance. When the objective of the evaluation is sensitivity, specificity, and precision, the best performance was achieved using 20% of all the features calculated in this study. The results for the metrics sensitivity and accuracy in the analysis of individuals with PD and HY = 1 were superior to the results found when we used all individuals with PD in this study. The obtained result indicates that objective methods can be applied in the evaluation of PD. The statistical data from this study are in agreement with the literature. The use of computational and objective methods aids in evaluating the signal of the inertial sensors of the individual performing the hand at rest task, while mitigating the effect of the variations of the symptoms. The technique proposed in this study allows for the automatic classification of individuals and can be used to aid in diagnosing patients with suspected PD, even in the initial stages of the disease.

## Methods

### Ethical aspects

This study was approved by the Human Research Ethics Committee (HERC—nº 270.782) of the Universidade Federal de Uberlândia, and by the National Ethics Research Committee (NERC—nº 361.526) of the National Health Council.

### Participants

The subjects with Parkinson’s disease were recruited from the ambulatory facility of the Hospital de Clínicas of Uberlândia of the Federal University of Uberlândia (HCU-UFU), at the physiotherapy clinic of the Federal University of the Triângulo Mineiro (Uberaba), and at the Parkinson association of the Triângulo Mineiro in Uberlândia.

Twenty-seven individuals from both genders were enrolled in this study, across the age group of 50 years or over. From these, 12 individuals were healthy and 15 diagnosed with PD (Table 4), in the initial stage of PD (stages 1 and 2 of the Hoehn and Yahr score (HY) [10]). The participants were divided into 2 groups, with the first group being made up of individuals with PD ${(S}_{\mathrm{PD}}$), and the second group of the healthy individuals (${\mathrm{S}}_{\mathrm{H}}$ = 12 individuals).

**Table 4:** Demographic characteristics of the participants of this study

Characteristics	*S*_PD_	*S*_H_
Number of subjects	15	12
Age (years) (mean ± SD)	65.3 ± 9.1	60.1 ± 6.1
Gender/number of subjects	M/8—F/7	M/4—F/8
Hand Analyzed /number of subjects	R/9—L/6	R/12

*M* male, *F* female, *R* right, *L* left, *SD* standard deviation

In this study, two analyses were performed. Initially, the classification between groups was carried out with healthy individuals and all individuals with PD ${(S}_{\mathrm{PD}}\hspace{0.17em}=$ 15 individuals). The second analysis consisted of the classification between healthy individuals and the group composed of individuals with PD with HY = 1 ${(S}_{\mathrm{PD}}=$ 11 individuals). The data collection was performed during the period “ON” of the individuals with PD. The subjects with PD were diagnosed by a neurologist.

Table 5 shows the tremor score according to the UPDRS scale part 3 (motor section) item 20 (tremor at rest) and the HY score of all PD volunteers.

**Table 5:** Score of UPDRS (part 3 item 20) and HY score of all PD volunteers across the study population

Volunteer	UPDRS (part 3, item 20)	Score HY
1	1	1
2	0	1
3	3	2
4	1	1
5	0	1
6	2	2
7	2	2
8	0	1
9	1	1
10	1	1
11	0	1
12	1	1
13	1	1
14	2	2
15	0	1

### Data collection

Data collection was performed using the device TREMSEN (Precise Tremor Sensing Technology, INPI: BR 10 2014 023282 6) developed by researchers from the Center for Innovation and Technological Evaluation in Health [Núcleo de Inovação e Avaliação Tecnológica em Saúde (NIATS)] from the Federal University of Uberlândia. TREMSEN is composed of a gyroscope (L3GD20H, STMicroelectronics, Switzerland), an accelerometer and a magnetometer. The sensibility of the gyroscope, accelerometer, and of the magnetometer was configured to ± 245°/s, ± 2 g and ± 2 gauss respectively, in accordance with the studies of [29].

The signals collected were digitalized at 50 Hz, using a microcontroller (Atmel SAM3X8E ARM Cortex-M3) with a 12-bit resolution digital/analog converter. The data were stored on a laptop by means of serial communication, the software used was developed for TREMSEN in C# (Microsoft). The data were stored in text format and processed by a custom code written in R-Studio.

**Fig. 3:**
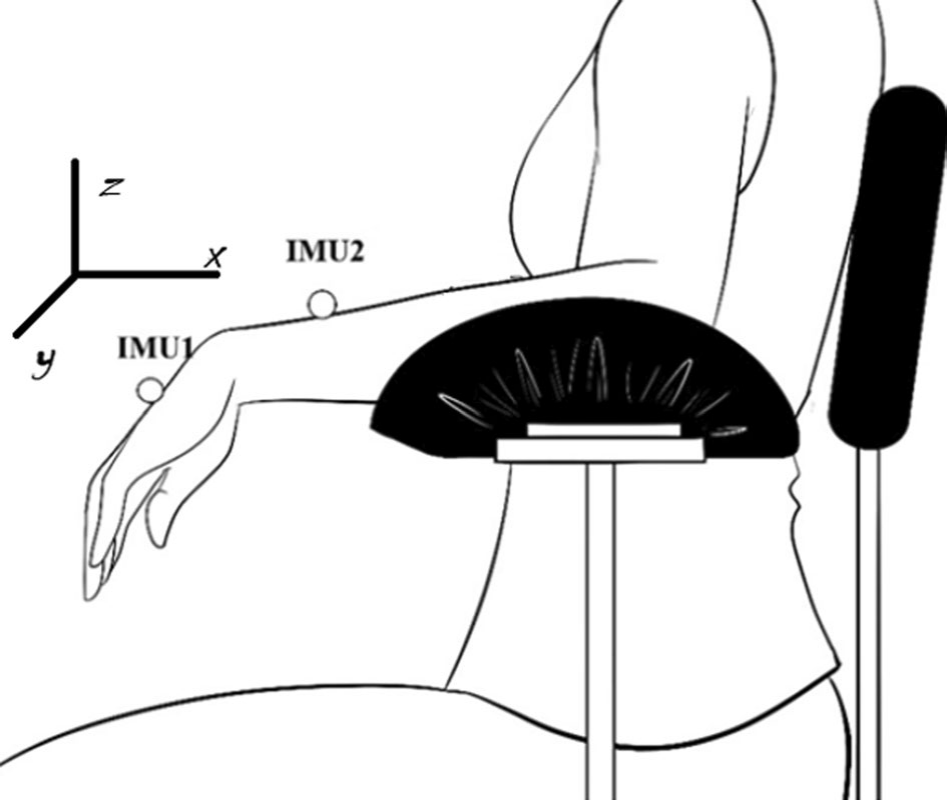
Positioning of the subject and inertial sensor unit, as well as the orientation of inertial sensor axes

Parkinson’s disease generates a complex set of movements, which is notorious for producing uncontrollable jerking movements of the forearm, wrist, and hand. Due to this fact, two sets of Inertial Measurement Unit (IMUs) were used. The IMU 1 was positioned on the back of the hand, the IMU 2 was positioned on the back of forearm, between 3 and 4 cm from the wrist joint, with the direction of the axes oriented according to Fig. 3. On the healthy individuals and individuals with PD without showing obvious signs of tremor (all individuals with PD with HY = 1), the inertial sensor was positioned on the dominant hand (Fig. 3), while on the individuals with PD and tremor, the sensor was positioned on the hand most affected by the tremor (four individuals with PD with HY = 2).

The data were collected from the participants in accordance with [29]. The subjects maintained the wrist at rest and pendant for a short period, the forearm supported on a rest with the hand pendant, forearm in pronation, and the palm of the hand facing downwards. The measurements were collected three times, with intervals of 60 s, where each collection was comprised of 15 s.

### Signal preprocessing

The signals were band-pass-filtered between 1 and 16 Hz to remove low- and high-frequency artifacts [34].

The start and end of tasks were marked manually by pressing a push-button, generating a pulse of 15 s (manual pulse). Following this, the resultant of the 3 axes of the accelerometer, gyroscope, and magnetometer sensors were calculated, using Eq. (1):

1 $$ R = \sqrt {x^{2} + y^{2} + z^{2} } , $$

where *x, y,* and *z* are the measurements from the sensors along their respective axes and *R* is the resultant.

For the removal of linear trends, the value of *R* was subtracted from its mean. The resulting signal was used to calculate the features.

### Extraction of features

The features used in this study are described in Table 6.

**Table 6:** Features extracted from the signals

Feature	Source of the features	Definition
Root mean square (RMS)	[35–38]	$RMS= \sqrt{\frac{1}{N}\sum_{n=1}^{N}{x(n)}^{2}}$ where $N$ is the number of elements of $X$ ($X=\{{x}_{1},{x}_{2},\dots ,{x}_{n}\}); x\left(n\right)$ is the $n$th element
Peak	[35]	Maximum value of the signal
Mean absolute value (MAV)	[35, 36, 39]	The patterns are organized into windows and the average value of each window is used as the value of the feature $MAV=\frac{1}{S}{\sum }_{m=1}^{S}\left|\left.{X}_{m}\right|\right.$ S is the number of samples per window; ${X}_{m}$ is the m-th sample of the window
Mean absolute value of the first difference (MAVFD)	[35, 40, 41]	$MAVFD=\frac{1}{N-1}\sum_{n=1}^{N-1}\left|x\left(n+1)-\right.\right.\left.x \left(n\right)\right|$
Mean absolute value of the second difference (MAVSD)	[35, 40]	$MAVSD=\frac{1}{N-2}\sum_{n=1}^{N-2}\left|x\left(n+2)-\right.\right.\left.x \left(n\right)\right|$
Mean frequency (FMEAN)	[35, 36, 38, 42, 43]	$FMEAN=\frac{\sum_{n=1}^{N}{(P}_{n}\left(n\right)*{f}_{n}(n))}{\sum_{n=1}^{N}{P}_{n}\left(n\right)}$ where ${P}_{n}$ is the Power spectrum; ${f}_{n}$ is the vector frequency of ${P}_{n}$; $N$ is the number of samples
Zero crossing (ZC)	[35–37, 39]	Computes how many times the signal crosses zero
Peak frequency (FPEAK)	[42–44]	FPEAK is a frequency at which the maximum power occurs FPEAK = maximum(${P}_{n}$)
Median frequency (F50)	[35, 36, 38, 42–44]	$\sum_{n=1}^{F50}{P}_{n}\left(n\right)=\sum_{F50}^{N}{P}_{n}\left(n\right)=\frac{1}{2}*\sum_{n=1}^{N}{P}_{n}\left(n\right)$
Frequency for which 80% of the total power of *P*_*n*_ is below this value (F80)	[43, 45]	$\sum_{n=1}^{F80}{P}_{n}\left(n\right)=0.8*\sum_{n=1}^{N}{P}_{n}\left(n\right)$
Power in frequency band 3.5–7.5 Hz (*Power3.5_7.5*)	[46]	$Power3.5\_7.5=\sum_{{f}_{n}=3.5}^{{f}_{n=7.5}}{P}_{n}\left(n\right)$
Approximate entropy (ApEn)	[35, 37, 45, 47, 48]	According to [48]: • For a time series of sample *N {u(1), u(2), u(3)…u(N)}* given *m,* forms sequences of vectors *x(1)* through *x(N-M* + *1)*, defined by *x(i)* = {*u(i), u (i* + *1),…, u (i* + *m—1)*}, *i* = *1,…, N—m* + *1*; • Compute the distance between the vectors *x(i)* and *x(j)* defined as the maximum difference between each element of the vectors (*d[x(i), x(j)]*); • For each *i* ≤ *N-m* + *1*, compute ${C}_{i}^{m}(r)$, which is defined as: ($number of j such as d[x\left(i\right), x\left(j\right)]\le r)/(N-m+1)$; • Define: ${C}^{m}\left(r\right)={\left(N-m+1\right)}^{-1}{\sum }_{i=1}^{N-m+1}ln{C}_{i}^{m}\left(r\right)$; • The approximated entropy is defined by: $ApEn\left(m,r,N\right)={C}^{m}\left(r\right)-{C}^{m+1}\left(r\right)$ where *m* is the length of the comparative window; *r* is the tolerance; *ln* is the natural logarithm
Fuzzy entropy (FuzzyEn)	[35, 37, 49]	According to [50, 51]: • For a time series of sample *N {u(1), u(2), u(3)…u(N)}* given *m,* forms sequences of vectors *x(1)* through *x(N-M* + *1)*, defined by *x(i)* = {*u(i), u (i* + *1),…, u (i* + *m—1)*}, *i* = *1,…, N—m* + *1*; • Compute the similarity degree between the vectors *x(i)* and *x(j)* defined by a fuzzy function: ${d}_{[x\left(i\right),x\left(j\right)]}^{m}=\mu ({d}_{ij}^{m},r$), where ${d}_{ij}^{m}$ is the maximum difference between each element of the vectors; • For each vector *x(i)* average all the similarity degree of its neighboring vectors (*i ≠ j*); • For each *i* ≤ *N-m* + *1*, compute ${P}_{i}^{m}(r)$, which is defined as: ${P}_{i}^{m}\left(r\right)={\left(N-m+1\right)}^{-1}\sum_{j=1,j\ne i}^{N-m}{d}_{[x\left(i\right),x\left(j\right)]}^{m}$; • Define: ${P}^{m}\left(r\right)={\left(N-m\right)}^{-1}{\sum }_{i=1}^{N-m}{P}_{i}^{m}\left(r\right)$ and ${P}^{m+1}\left(r\right)={\left(N-m\right)}^{-1}{\sum }_{i=1}^{N-m}{P}_{i}^{m+1}\left(r\right)$; • The fuzzy entropy is defined by: $FuzzyEn\left(m,r,N\right)={lnP}^{m}\left(r\right)-{lnP}^{m+1}\left(r\right)$
Variance (*VAR*)	[35–37]	${\mathrm{VAR}=\sigma }^{2}=\frac{1}{N-1}{\sum }_{n=1}^{N}\left(x\left(n\right)-\overline{x }\right){ }^{2}$ $\overline{x }$–average of the samples
Range (*RANGE*)	[35, 52]	Difference between the maximum and minimum value of the signal
Range interquartile (*IntlA*)	[35, 53, 54]	$\mathrm{IntIA}= {Q}_{3}-{Q}_{1}$ ${Q}_{3}$—third quartile; ${Q}_{1}$—first quartile
Skewness (*Skewness*)	[41, 45, 55]	$\mathrm{Skewness}=\frac{\frac{1}{n}\sum_{n=1}^{N}{(x\left(n\right)-\overline{x })}^{3}}{{\sigma }^{3}}$
Kurtosis (*Kurtosis*)	[41, 45, 55]	$\mathrm{Kurtosis}=\frac{\frac{1}{n}\sum_{n=1}^{N}{(x\left(n\right)-\overline{x })}^{4}}{{\sigma }^{4}}$

108 features were extracted from each participant, considering 18 features for each sensor (accelerometers, gyroscopes and magnetometers from IMUs 1 and 2).

### Features selection

The features used to feed the classifiers were selected using the software R package, CORElearn, and the attrEval function using the ReliefF estimator and with *k* = 1. The classifiers were fed with 10% of the most important features, then 20% of the most important features, and so on, in incremental steps of 10% until all the features are added for calculating the metrics (sensitivity, specificity, precision, and accuracy).

### Classifiers

After the extraction of the features, the data were normalized using the *Z*-score technique. This technique is used in order that each feature is presented on the same scale in a dimensionless form. The equation for the *Z*-score is given by Eq. (2):

2 $$ {\text{ZS}} = \left( {\frac{{{\text{value}} - \mu }}{\sigma }} \right), $$

where $\mu$ is the mean of the features and $\sigma$ its standard deviation.

The classifiers used were:

- KNN;
- SVM;
- RF;
- NB.

These classifiers were chosen due to their use in various studies [56, 57].

Simulations were performed to identify the best parameters for the classifiers. In the KNN method, there exist proposals in the literature for the adoption of the K value, as being the square root of the size of the training set, but in our simulations, the value of *k* = 3 offered the best results. In regard to the remaining classifiers, the best results for Random Forest were obtained using 120 trees, for the SVM, the polynomial kernel will be used, and for the classifier Naive Bayes, the kernel (normal) predictor will be used.

### Statistical analysis

To evaluate the performance of a classifier, a confusion matrix (Table 7) was built considering the true positives (TP), false positives (FP), true negatives (TN), and false negatives (FN). Metrics for performance, sensitivity, specificity, precision, and accuracy (Eqs. 3–6, respectively), were computed for each classifier

3 $$ {\text{Sensitivity}} = \frac{{{\text{TP}}}}{{{\text{TP}} + {\text{FN}}}} $$

4 $$ {\text{Specificity}} = \frac{{{\text{TN}}}}{{{\text{TN}} + {\text{FP}}}} $$

5 $$ {\text{Precision}} = \frac{{{\text{TP}}}}{{{\text{TP}} + {\text{FP}}}} $$

6 $$ {\text{Accuracy}} = \frac{{{\text{TP}} + {\text{TN}}}}{{{\text{TP}} + {\text{TN}} + {\text{FP}} + {\text{FN}}}}. $$

**Table 7:** Confusion matrix

	Positive result	Negative result
Actual positive	TP	FN
Actual negative	FP	TN

The data from the subjects were randomly divided into training (about 50%) and testing (about 50%) sets. Thus, the data from the 14 subjects were allocated to training and the data from the remaining 13 subjects were reserved for testing. We used tenfold cross-validation to evaluate the classification performance. The R Project for Statistical Computing was used to conduct data analyses.

## Supplementary Information

**Additional file 1: Figure S1.** A typical accelerometer signal from one of the volunteers. Where: a) Signal on the X-axis. b) Signal on the Y-axis. c) Signal on the Z-axis. d) Resultant signal (blue) and manual pulse (red). **Figure S2.** A typical gyroscope signal from one of the volunteers. Where: a) Signal on the X-axis. b) Signal on the Y-axis. c) Signal on the Z-axis. d) Resultant signal (blue) and manual pulse (red). **Figure S3.** A typical magnetometer signal from one of the volunteers. Where: a) Signal on the X-axis. b) Signal on the Y-axis. c) Signal on the Z-axis. d) Resultant signal (blue) and manual pulse (red).

## Data Availability

The datasets generated in the current study are not publicly available due to the ethical restrictions preventing public sharing of data. A non-identified set may be requested after approval from the Review Board of the Institution. Requests for the data may be sent to the corresponding author.

## Abbreviations

PD: Parkinson disease
RF: Random forest
SVM: Support vector machine
KNN: K-nearest neighbor
NB: Naive Bayes
UPDRS: Unified Parkinson’s Disease Rating Scale
IMU: Inertial measurement unit
HERC: Human Research Ethics Committee
NERC: National Ethics Research Committee
HCU-UFU: Hospital de Clínicas of Uberlândia of the Federal University of Uberlândia
SPD: Parkinson disease group
SH: Healthy group
M: Male
F: Female
R: Right
L: Left
SD: Standard deviation
TREMSEN: Precise Tremor Sensing Technology
NIATS: Center for Innovation and Technological Evaluation in Health
TP: True positives
FP: False positives
TN: True negatives
FN: False negatives
RMS: Root mean square
MAV: Mean absolute value
MAVFD: Mean absolute value of the first difference
MAVSD: Mean absolute value of the second difference
FMEAN: Mean frequency
ZC: Zero crossing
FPEAK: Peak frequency
F50: Median frequency
Power3.5_7.5: Power in frequency band 3.5–7.5 Hz
ApEn: Approximate entropy
FuzzyEn: Fuzzy entropy
VAR: Variance
IntlA: Range interquartile
