# Supplementary material for: Discrimination between healthy and patients with Parkinson’s disease from hand resting activity using inertial measurement unit
Source: Biomed Eng Online. 2021 May 22;20:50. doi: 10.1186/s12938-021-00888-2 (PMC8141164; doi:10.1186/s12938-021-00888-2)
Supplement: Supplementary file 1 — Additional file 1: Figure S1. A typical accelerometer signal from one of the volunteers. Where: a) Signal on the X-axis. b) Signal on the Y-axis. c) Signal on the Z-axis. d) Resultant signal (blue) and manual pulse (red). Figure S2. A typical gyroscope signal from one of the volunteers. Where: a) Signal on the X-axis. b) Signal on the Y-axis. c) Signal on the Z-axis. d) Resultant signal (blue) and manual pulse (red). Figure S3. A typical magnetometer signal from one of the volunteers. Where: a) Signal on the X-axis. b) Signal on the Y-axis. c) Signal on the Z-axis. d) Resultant signal (blue) and manual pulse (red). [file 12938_2021_888_MOESM1_ESM.docx]

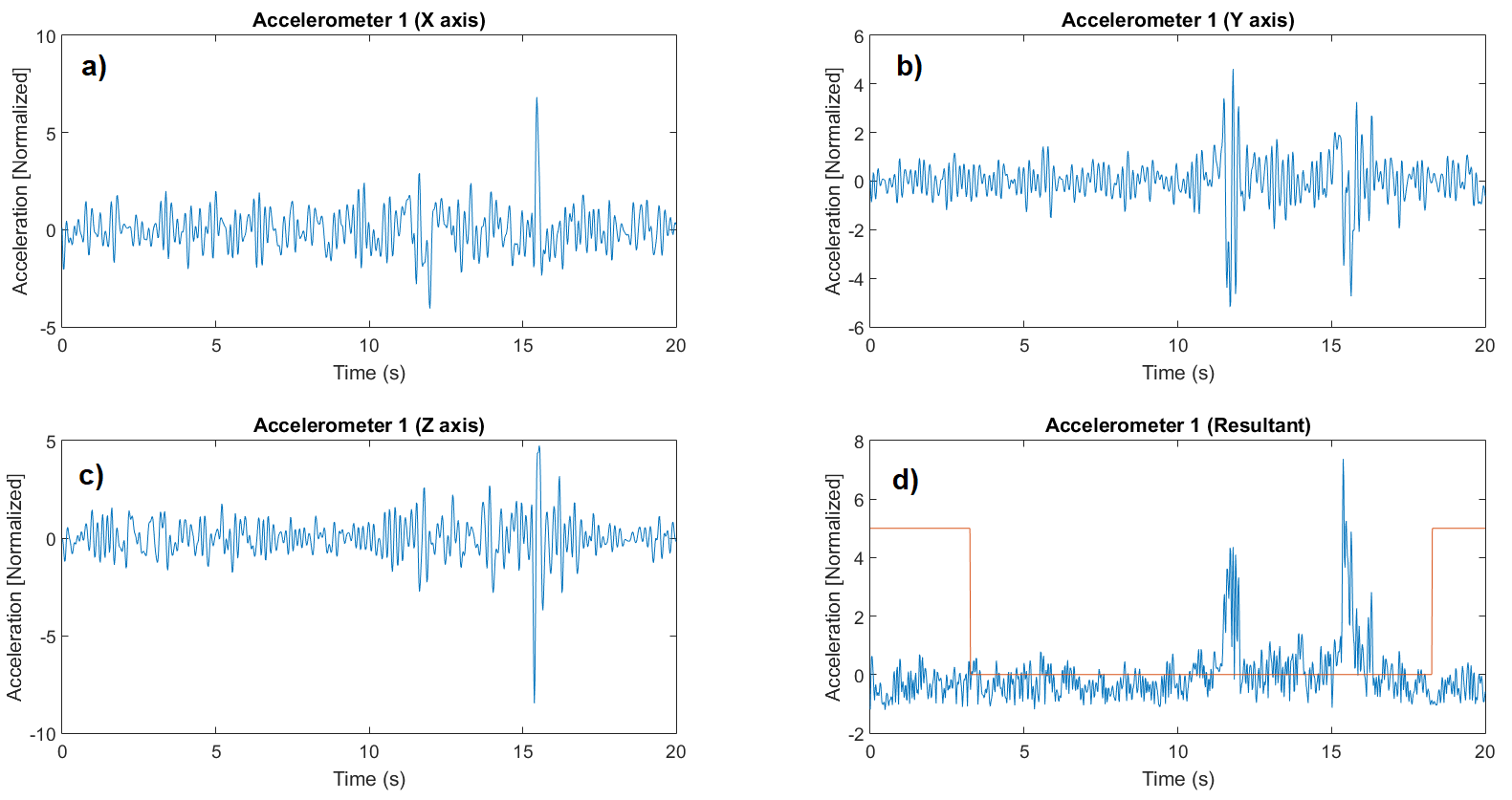


**Figure S1** – A typical accelerometer signal from one of the volunteers. Where: a) Signal on the X-axis. b) Signal on the Y-axis. c) Signal on the Z-axis. d) Resultant signal (blue) and manual pulse (red).


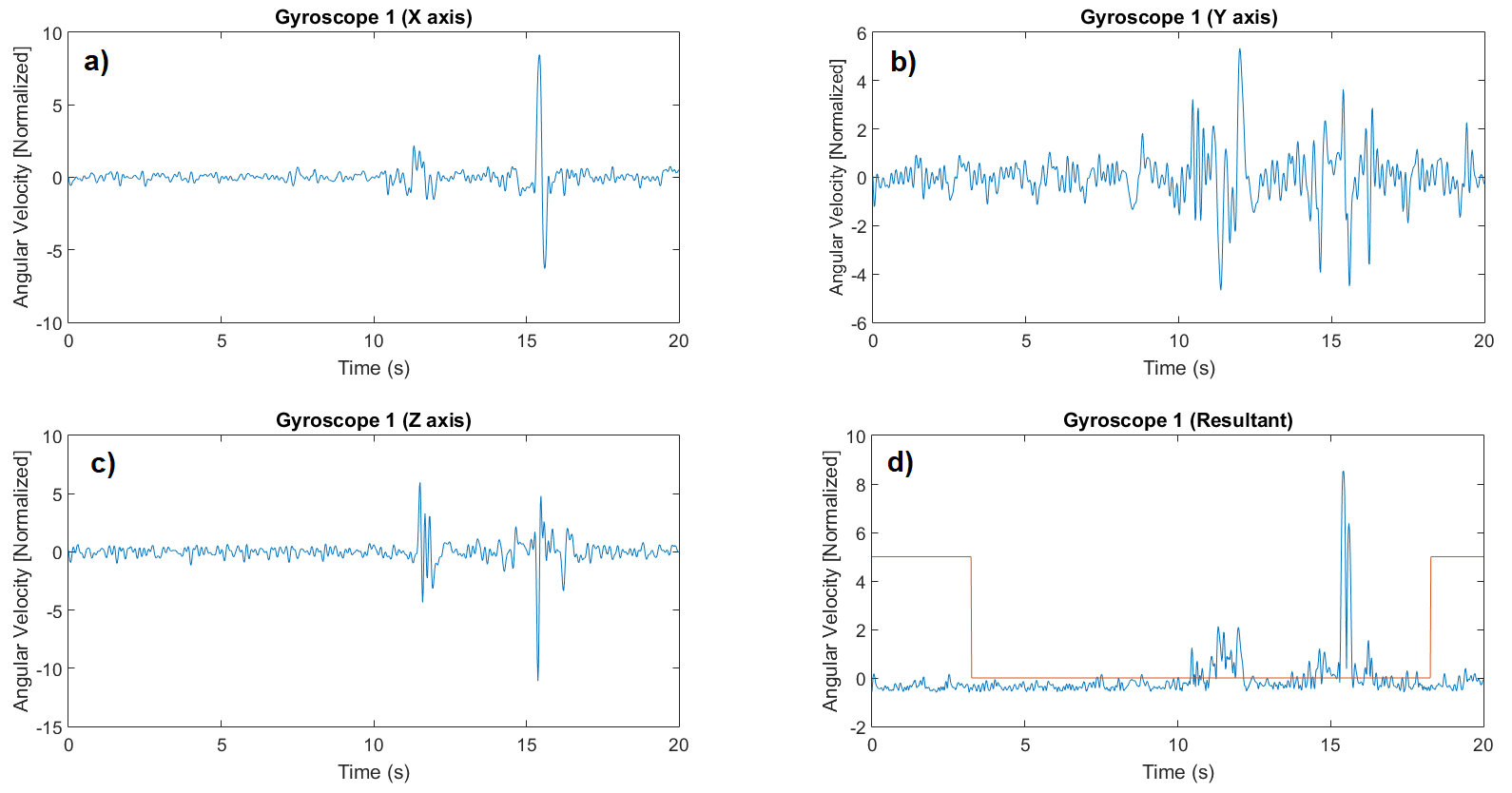


**Figure S2** – A typical gyroscope signal from one of the volunteers. Where: a) Signal on the X-axis. b) Signal on the Y-axis. c) Signal on the Z-axis. d) Resultant signal (blue) and manual pulse (red).


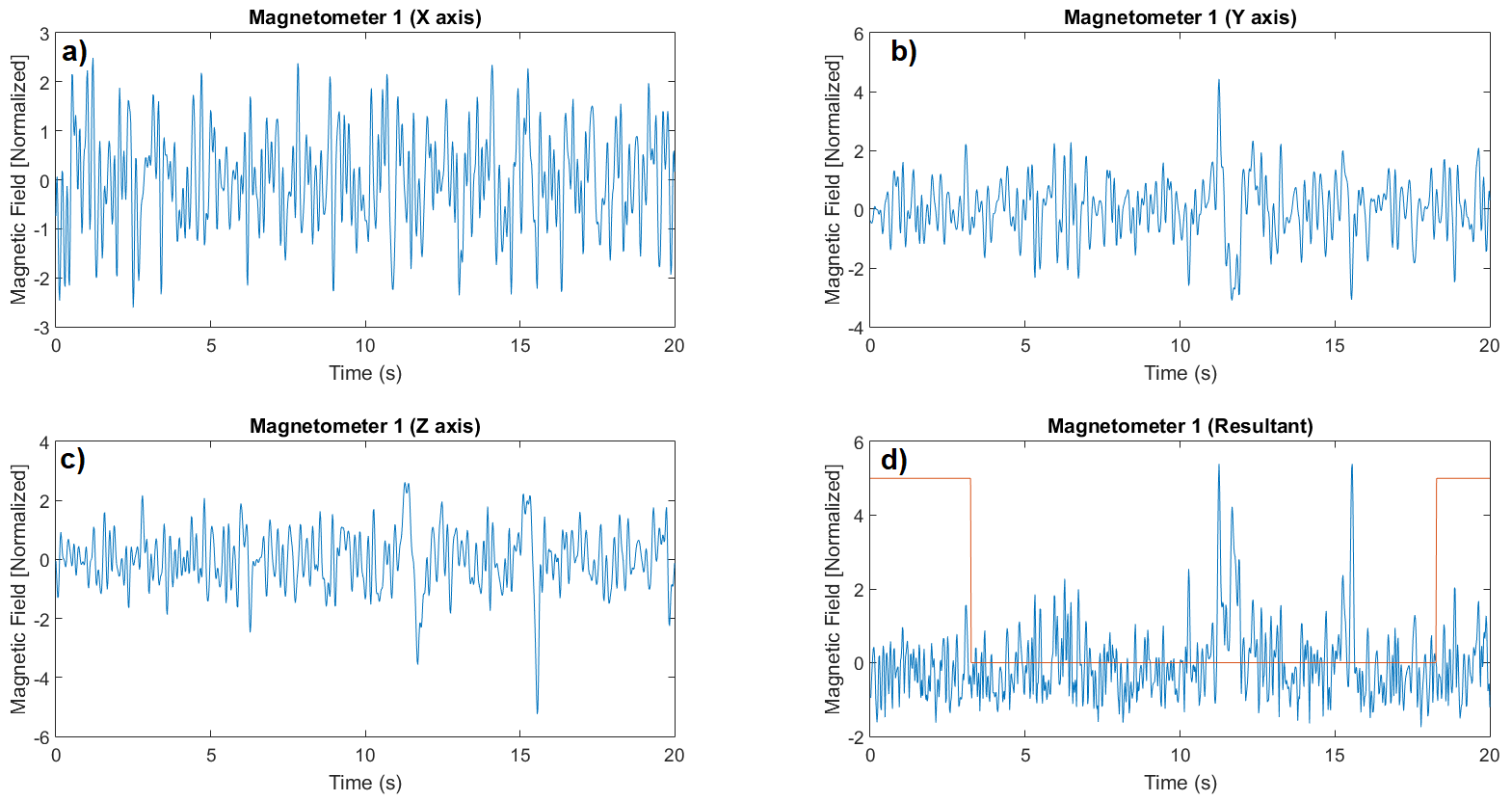


**Figure S3** – A typical magnetometer signal from one of the volunteers. Where: a) Signal on the X-axis. b) Signal on the Y-axis. c) Signal on the Z-axis. d) Resultant signal (blue) and manual pulse (red).
